# Supplementary figures and images for: Identification and Characterization of Malate Dehydrogenases in Tomato (Solanum lycopersicum L.)
Source: Int J Mol Sci. 2022 Sep 2;23(17):10028. doi: 10.3390/ijms231710028 (PMC9456053; doi:10.3390/ijms231710028)

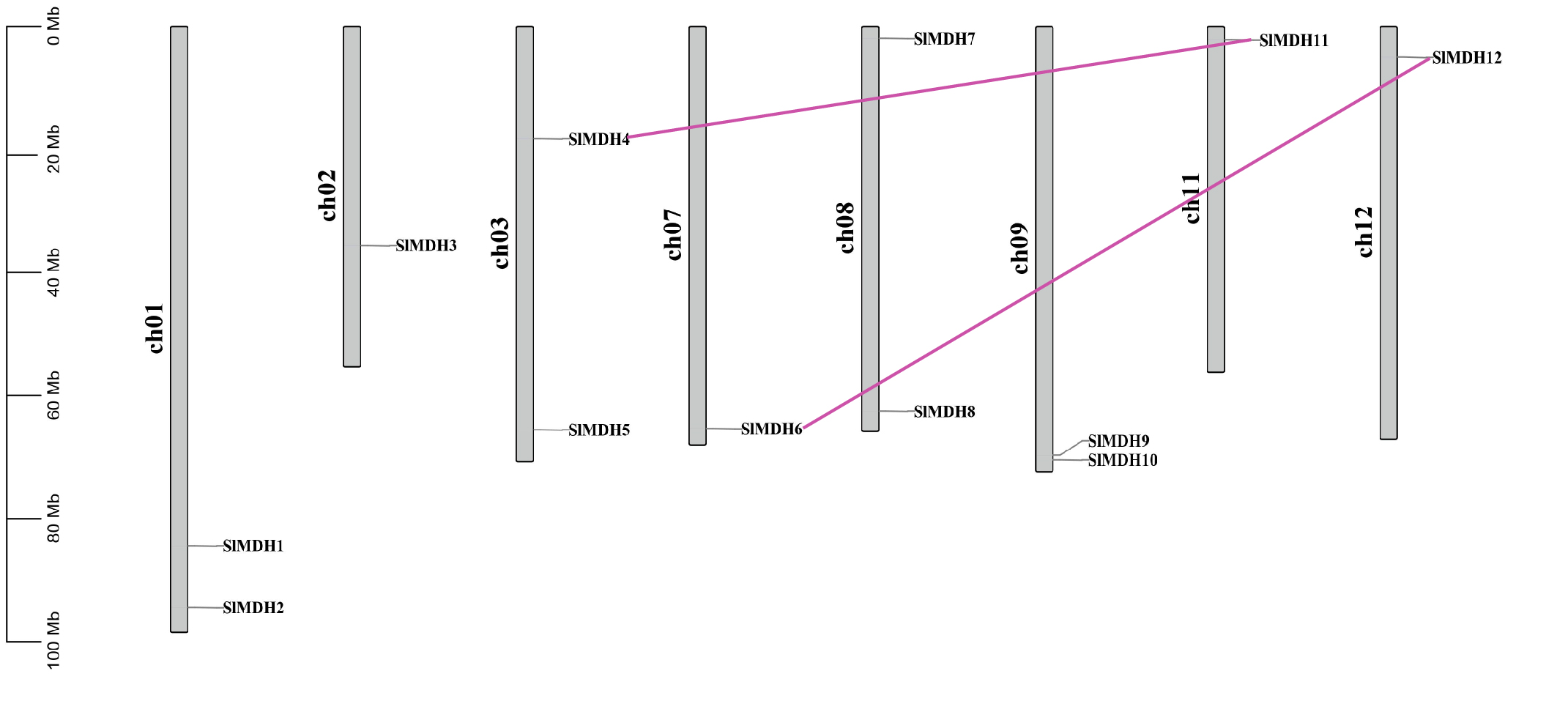

Supplement: Supplementary file 1 [file ijms-23-10028-s001.zip › ijms-1767606-supplementary/Figure S1.jpg]
